# Can HCV Core Ag Testing Replace the Routine Screening Assays in Patients on Hemodialysis?

**DOI:** 10.5812/hepatmon.16371

**Published:** 2014-07-01

**Authors:** Behzad Einollahi, Neda Raeessi, Fereshteh Raeessi

**Affiliations:** 1 Nephrology and Urology Research Center, Baqiyatallah University of Medical Sciences, Tehran, IR Iran; 2 Atherosclerosis Research Center, Baqiyatallah University of Medical Sciences, Tehran, IR Iran; 3 Clinical Research Center, Azad University of Pharmacy, Tehran, IR Iran

**Keywords:** Hepatitis B Virus, Hepatitis C Virus

Dear Editor

We have recently read with great interest the article published in your most valuable journal entitled “Hepatitis C virus infection rate among seronegative hemodialysis patients screened by two methods: HCV core antigen and polymerase chain reaction” by Moini et al. (1). This study focused its message on determination of the prevalence of hepatitis C virus (HCV) infection in seronegative hemodialysis (HD) patients using two methods of HCV core antigen (Ag) testing by ELISA assay, and the real time polymerase chain reaction. The authors have concluded that HCV core Ag testing could be used as a sensitive method for HCV infection screening in patients on HD (1).

We agree that patients on long-term HD are at higher risk of acquiring HCV infection (2, 3). Despite administration of recombinant erythropoietin, HCV transmission is still being reported among patients on HD (4, 5). Undoubtedly, HCV screening is crucial for patients on HD since these population is at increased risk of infection and moreover it easily spreads the infection through dialysis units (6). HCV infection is routinely diagnosed by measuring anti-HCV antibody whereas the antibody is undetectable in the first four to six weeks of infection, the so-called, window period (7). Furthermore, there is a blunted antibody response in patients on HD due to depressed immune system (1). Kidney Disease Improving Global Outcomes (KDIGO) has recommended the use of nucleic acid amplification technology (NAT) in detection and evaluation of HCV in chronic kidney disease (8). By utilizing NAT, some problems emerge: the kits are frequently unavailable; considerable skill is required; there is limited reproducibility; and most importantly, the costs are steep (7). Some studies in the general population have pointed out the importance of HCV core Ag detection as an alternative to NAT for early diagnosis of infection as well as predicting and monitoring the response to therapy (9); however few studies concerning its efficacy in patients on long-term HD exist (7, 10).

We totally concur with the authors that HCV core Ag may be an accurate marker for early detection and virological monitoring of HCV infection in patients on HD. Owing to the low cost and its technical easiness, this assay can be employed for routine screening of patients on HD; however, more studies with larger sample size are still required to effectively establish HCV core Ag test as a preferred assay for the early diagnosis of HCV infection in patients on HD.
